# Multimodality Cardiovascular Imaging for Totally Video-Guided Thorascopic Cardiac Surgery

**DOI:** 10.31083/j.rcm2505181

**Published:** 2024-05-21

**Authors:** Qin Jiang, Keli Huang, Lixue Yin, Bo Zhang, Yiping Wang, Shengshou Hu

**Affiliations:** ^1^Department of Cardiac Surgery, Sichuan Provincial People’s Hospital, Affiliated Hospital of University of Electronic Science and Technology, 610072 Chengdu, Sichuan, China; ^2^Ultrasound in Cardiac Electrophysiology and Biomechanics Key Laboratory of Sichuan Province, Sichuan Provincial People’s Hospital, University of Electronic Science and Technology of China, 610072 Chengdu, Sichuan, China; ^3^Department of Operating Room, Sichuan Provincial People’s Hospital, Affiliated Hospital of University of Electronic Science and Technology, 610072 Chengdu, Sichuan, China; ^4^Department of ICU, Sichuan Provincial People’s Hospital, Affiliated Hospital of University of Electronic Science and Technology, 610072 Chengdu, Sichuan, China; ^5^Department of Cardiac Surgery, Fuwai Hospital, National Center for Cardiovascular Disease, Chinese Academy of Medical Sciences and Peking Union Medical College, 100037 Beijing, China

**Keywords:** minimally invasive cardiac surgery, totally video-guided thoracoscopic cardiac surgery, multimodality cardiovascular imaging

## Abstract

Totally video-guided thorascopic cardiac surgery (TVTCS) represents one of the most minimally invasive access routes to the heart. Its feasibility and safety can be guaranteed by an experienced surgeon with skilled operative techniques under the guidance of a video signal via thoracoscopy and the imaging from transesophageal echocardiography. At present, this surgical approach has been applied for atrioventricular valve disease, atrial septum defects plus and partial anomalous pulmonary venous drainage, cardiac tumors, hypertrophic obstructive cardiomyopathy, aortic valve disease, and atrial fibrillation. Multimodality cardiovascular imaging, including echocardiography, X-ray, computed tomography (CT), magnetic resonance imaging (MRI) and cardiac catheterization, provides morphologic characteristics and function status of the cardiovascular system and a comprehensive view of the target anatomy. In this review, the benefits of multimodality cardiovascular imaging are summarized for the clinical practice of TVTCS, including the preoperative preparation, intraoperative guidance and postoperative supervision. The disease categories are also individually reviewed on the basis of multimodality cardiovascular imaging, to ensure the feasibility and safety for TVTCS. Cardiovascular imaging technologies not only confirm who is a candidate for this surgical technique, but also provide technical support during the procedure and for postop follow to assess the clinical outcomes. Multimodality cardiovascular imaging is instrumental to provide the requirements to solve the problems for conduction of TVTCS; and to provide individualized protocols with high-resolution and real-time dynamic imaging fusion.

## 1. Introduction

Minimally invasive cardiac surgery (MICS) is developing at a rapid pace due to the innovation of medical instruments. Totally video-guided thorascopic cardiac surgery (TVTCS) represents one of the most minimally invasive approaches in cardiac surgical community [1]. Cardiac surgeons have acquired specialized skills to conduct cardiac procedures under the guidance of a video field projected from high-resolution transition signals with thoracoscopy with and without cardiopulmonary bypass (CPB). The disease spectrums covered by this technique range from mitral or tricuspid valve dysfunction, congenital heart defects such as atrial septum defects (ASD), incomplete endocardial cushion defects, partial anomalous pulmonary venous drainage, cardiac tumors, and hypertrophic obstructive cardiomyopathy (HOCM) with left ventricular outflow tract obstruction [2]. This minimal port-access approach has been successfully applied to rescue technical complications including occluder device failure and migration during percutaneous closure of ASDs [3], failed mitral valve clip device implantation [4] and paravalvular leakage after primary mitral valve replacement [5]. Combined cardiac procedure using TVTCS have resulted in less bleeding than from staged procedures [6]. Moreover, TVTCS reduces s postoperative systemic inflammation compared to a median sternotomy (MS) for an ASD [7] and for mitral valve diseases [8].

**Fig. 1.**
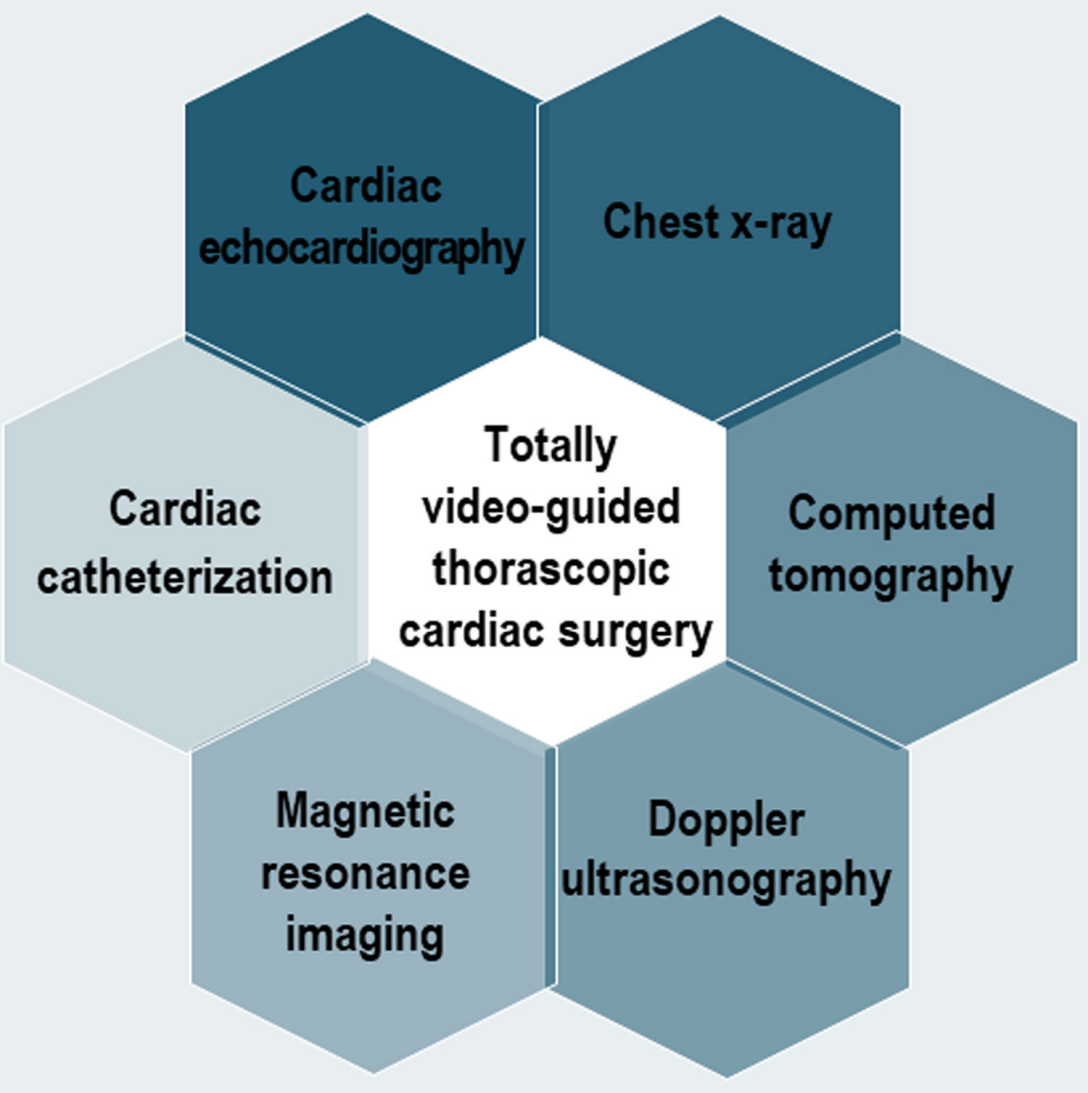
**Multimodality cardiovascular imaging for totally video-guided thorascopic cardiac surgery**.

Nevertheless, TVTCS can still result in unexpected risks and pitfalls. Multimodality cardiovascular imaging (MCI) is now used in increasing frequency in cardiac surgical procedures. It consists of cardiac echocardiography, chest X-ray, computed tomography, doppler ultrasonography, magnetic resonance imaging, and cardiac catheterization (Fig. 1). MCI for TVTCS is distinct from the traditional MS approach. It is rarely used for the evaluation of myocardial ischemia and cardiac tumors compared with that of traditional MS approaches. But for clarifying the feasibility and safety of TVTCS, it can be used to avoid intraoperative complications [9]. It has been used to exclude patients for TVTCS who are better suited for the traditional MS approach.

## 2. Multimodality Cardiovascular Imaging (MCI)

### 2.1 Cardiac Echocardiography

Echocardiography is the first-line imaging modality, which provide accurate diagnosis on structural heart diseases including valvular heart disease and congenital heart defects and help to decide which cases are more suitable for percutaneous interventions such as defect occlusion and valve replacement if indicated by echocardiography imaging. Transthoracic echocardiography (TTE) is economical and widely available, should be recommended for all the patients undergoing cardiac surgery at admission, and following discharge. The severity of cardiac systolic dysfunction and pulmonary artery hypertension are pivotal parameters of exclusion criteria for TVTCS to avoid the possibility of inability to wean from CPB.

Transesophageal echocardiography (TEE) is essential for patients during TVTCS and provides vital information prior to, during, and following the procedure. Echocardiographic visualization also helps during the positioning of femoral artery and veinous cannulation for establishing CPB. The coexistence of aortic insufficiency with an effective regurgitate orifice area (EROA >4.0 ${\mathrm{cm}}^{2}$) is a relative contraindication for TVTCS technique since adequate antegrade cardioplegia cannot be safely delivered to ensure optimal myocardial protection during aortic clamping. Specific parameters from TEE measurements after induction of anesthesia could further validate the diagnosis and more precisely evaluate the hemodynamic parameters such as tricuspid annular velocity [10]. For isolated tricuspid valve surgery, the severity of valve regurgitation is evaluated preop to help determine the type repair to be performed [11]. Postoperatively, the performance of the repaired valve or prothesis valve is assessed. The timely evaluation of cardiac function and deairing the cardiac chambers is important for weaning from CPB.

### 2.2 Chest X-Ray

All patients should have routine anteroposterior and lateral Chest X-rays. A chest X-ray is helpful to select the suitable port access. The optimized layout of access-ports provides the most advantageous working angles, and facilitates the intraoperative manipulation of the surgical instruments in the limited space from the entrance to the cardiac cavity [12]. By convention TVTCS is performed from 3 cm main port-access sites on the intersection of the right anterior axillary line and the fourth intercostal space. Obese patients may present with a higher positioned diaphragm and caution should be used when placing the port at the anterior axillary line and the fifth intercostal space. In contrast, port access is more comfortable for the leaner and the underweight populations due to larger room in the intercostal space. The symmetrical heart mirror-image orientation is rare but should be kept in mind when the mini-thoracotomy access technique is performed from the left side of the chest [13]. Chest bedside X-ray imaging plays an essential role in detecting early postoperative complications such as re-expansion pulmonary edema after TVTCS [14].

### 2.3 Computed Tomography (CT)

A plain chest computed tomography (CT) scan is highly indicated if there is a history of lung diseases such as pleural effusions and infections. CT imaging could provide more accurate information on the morphology of the lungs and the pleura in addition to obtaining a past medical history of previous surgery and infections to avoid unnecessary injuries when performing the thoracotomy in case there are extensive adhesions. Loose pleural adhesions do not pose a challenge to the surgeon when entering the thoracic cavity. An experienced surgeon can handle dense adhesions and repair iatrogenic pulmonary perforations. Dissection of dense pleural adhesions and fibrosis prolong operative times and may lead to prolonged air leaks which delays the removal of chest drainage tubes. Severe thoracic wall and spine deformities could deter the enthusiasm for surgeons to perform TVTCS. But mild or moderate congenital thoracic wall deformities are not contraindicated for port-access surgical procedures [15]. CT is also valuable to provide anatomy characteristics for proper positioning of central venous catheters since the carina level on chest radiography was about 2 cm above the superior vena cava-right atrium junction [16]. Chest CT can also detect aorta calcification so as to avoid the embolic debris when placing the aortic cross clamp. Preoperative recognition of a porcelain aorta helps the surgeon to choose a non-atherosclerotic cannulation site or use an endoaortic balloon instead of the traditional Chitwood clamp [17]. Aortic CT angiography (CTA) could identify vulnerable plaques, kinking of iliac vessels, and small femoral artery diameters all of which are potential contraindications for TVTCS. CTA, through multiplanar reconstructions, could provide cross-sectional and three-dimension anatomy and show the geometry of the aorta especially if there is a suspicion of a coarctation of the descending aorta [18]. Severe iatrogenic complications such as aortic dissections or intramural hematomas during TVTCS complicated by retrograde femoral artery cannulation and ascending aortic cross-clamping can also be confirmed with a CTA examination [19]. When there are signs of ischemia on the electrocardiogram, myocardial perfusion deficiency or regional wall motion abnormalities under stress conditions [20], preoperative coronary CTA or angiography is obligatory to rule out a coronary artery stenosis [21]. Cardiac enhanced CT allows for the visualization of the cardiac cavity to evaluating post-procedural outcomes, especially for left atrium volume reduction during atrial fibrillation ablation [22].

### 2.4 Doppler Ultrasonography

Doppler ultrasonography is beneficial to obtain the morphology and flow-pattern of peripheral vessels such as the femoral artery, femoral vein, jugular vein and subclavian vein. Smaller caliber femoral arteries are not uncommon in young or slim individuals, this is especially true for females with rheumatic mitral stenosis. Under such circumstances, bilateral femoral artery cannulation with compatible sized cannulas can help to reduce vascular injuries [1]. Ascending aortic cannulation under the guidance of a puncture needle through a guidewire is the alternative to femoral artery cannulation due to calcification and thrombus [23]. Apart from a small caliber femoral artery, the underlying causes of cannulation difficulty or failure are due to anatomical deformity or variations of the femoral vein which preclude the establishment of CPB. The determination of blood-flow along inferior vena cava and femoral vein is imperative for excluding the feasibility of MICS in the patients with a past history of limb deep venous thrombosis, or Budd-Chiari syndrome [24]. Bilateral internal jugular vein cannulation is feasible during TVTCS for young individuals with a small lumen of the internal jugular vein [25]. Another approach to reduce the difficulty of cannulation of the jugular vein is the placement of bilateral subclavian vein cannulation [26].

### 2.5 Magnetic Resonance Imaging (MRI)

Cardiac magnetic resonance imaging (MRI) is considered to be the gold-standard imaging modality for noninvasive assessment of cardiac anatomy, motor function and myocardial substrate [27]. Left ventricular mass evaluation is a valuable tool to differentiate the diagnosis of large left and right ventricles secondary to mitral and tricuspid valve regurgitation from a dilated cardiomyopathy [28]. The integrative approach based on MCI is of paramount importance in the evaluation of dilated cardiomyopathy and provides incremental prognostic and therapeutic information [29]. Nevertheless, the kind of patients who have excessively dilated ventricules and decreased systolic function are usually contraindicated for TVTCS.

Three-dimensional late gadolinium enhancement MRI is used to evaluate the extent of ablation scar in patients undergoing hybrid atrial fibrillation (AF) ablation combined with thorascopic epicardial ablation with a percutaneous endocardial ablation [30]. The combination of cardiovascular MRI angiography and pulmonary perfusion allows for assessment of the anatomical stenosis of the pulmonary veins and its hemodynamic impact on the pulmonary parenchymal, which was conducive to evaluating the patency of the reestablished passage in such cases when performing the Warden procedure [31]. The esophageal thermal injury after interventional ablation for AF was confirmed by late gadolinium enhancement MRI [32]. Cardiac MRI should also be considered if there was complex cardiac and pulmonary malformation, such as the cases with extralobar sequestration and asymptomatic absence of pericardium in the patients with congenital heart defects [33].

### 2.6 Cardiac Catheterization

Coronary angiography is indicated for the patients with high risk of coronary heart disease, including older age (>50 years), hyperlipidemia, hyperglycemia, hypertension, and smoking [34]. Minimally invasive off-pump coronary artery bypass grafting should not be performed via thoracoscopy [35] but under CPB [36]. In the presence of moderate or severe pulmonary artery hypertension, right cardiac catheterization is essential for evaluating the possibilities of total closure of the atrial septal defect [37].

## 3. Cardiac Surgical Diseases

### 3.1 Mitral Valve and or Tricuspid Valve Diseases

Echocardiography not only provides the basic diagnosis on structural heart diseases, but provides important information on the size of the cardiac chambers for preoperative planning [38]. Mitral valve stenosis in young female adults can be performed with TVTCS, and is commonly associated with streptococcal infection [39]. Degenerative mitral regurgitation and mitral valve dysfunction secondary to congenital heart defects are another two areas for performing TVTCS. Two-dimesion echocardiography is recommended to acquire the characteristic findings of valvular and subvalvular abnormalities in rheumatic heart disease, including commissural fusion, leaflet thickening, and restricted leaflet mobility, with varying degrees of calcification [38]. Three-dimension echocardiography of live-multiplanar reconstruction function enhances the precision at real-time imaging of high temporal and spatial resolution and enables visualization of structures in multiple planes [40]. Three-dimensional TEE with dedicated mitral valve (MV) quantitation software is the reliable echocardiographic tool for the evaluation of the mitral annulus [41]. Echocardiography allowed for the findings of severe mitral valve thickening and calcification and to exclude patients who are not suitable candidates for TVTCS at the discretion of the surgeon. Tissue that remains in the cardiac cavity at the time of removing the diseased valve apparatus can result in a cerebral embolism. In contrast to echocardiography which lacks reproducibility and accuracy, cardiovascular magnetic resonance (CMR) is a better predictor of clinical outcomes and postsurgical left ventricular remodeling than echocardiography [42].

Mitral and tricuspid annulus size assessment is of utmost importance for the management of the patients with MV abnormalities regardless of the type of valve repair or replacement. The enlarged dimensions at the cardiac atrium and the expanded size of the MV annulus is advantageous for the surgeon to operate, but the reductant left atrium (LA) could limit visualization of the operative field. In cases of rheumatic MV stenosis, the replacement of diseased valve with an artificial prothesis is sometime difficult in the small MV annulus due to mitral annulus fibrosis, which could result in the placement of a smaller size prosthetic valve and the development of patient-prosthesis mismatch [43]. The unsuitable orientation of the strut, especially in bioprosthetic heart valves in could precipitate disruption of the left ventricular posterior wall when the MV apparatus is excessively removed [44]. On the contrary, restriction of mechanic valve leaflets due to excessive preservation of the sub-valvular apparatus is increasingly likely and results in acute mechanical prosthetic valve dysfunction. Mitral Valve replacement by TVTCS is a possibility for a high-risk patient following failed mitral valve valvuloplasty [45].

For concomitant tricuspid valve dysfunction secondary to MV disease, the dimensions of the right ventricle and pulmonary artery systolic pressure are to some extent reduced postoperatively in most patients [46]. Moderate tricuspid valve regurgitation or less-than-moderate regurgitation with annular dilatation (exceeding 40 mm in diameter) is still being recommended for prophylactic tricuspid annuloplasty repair to prevent progression of long-term regurgitation even if the risk of permanent pacemaker implantation is increased [47].

### 3.2 Congenital Heart Defect

ASD represents the main type of congenital heart defect undergoing TVTCS. Primary ASD is relatively less frequent than secundum ASD but it still could be done by the experienced surgeons who are good at mitral valve repair using several Carpentier techniques [48]. Persistent left-sided superior vena cava (SVC, PLSVC) is one of the common comorbidities of secundum ASD when there is a relatively small-sized right SVC in the presence of double vena cava drainage. The anatomy of PLSVC is diverse and portends requires a more complex corrective procedure. It should be highly suspected when an abnormal blood vessel exits on the left atrium (LA) especially between the left atrial appendage (LAA) and the left upper pulmonary vein. When in doubt, the diagnosis can be confirmed using TEE with agitated saline contrast injected into the LA [49]. CT and echocardiography show that the right-sided SVC empties into the right atrium, whereas the left-sided SVC shows typical drainage into the markedly dilated coronary sinus [50]. The detection of a PLSVC with CT perfusion imaging was also reported by differential myocardial perfusion imaging [51]. The choice of TVTCS in this population should be cautious because conventional manipulations lacking good cannulation drainage of the left internal jugular vein may jeopardize left cerebrum drainage and result in unilateral cerebral edema [52]. The practicability during TVTCS of the placement of bilateral jugular vein or subclavian vein cannulas need to be investigated in cases of PLSVC depending on the efficacy of the drainage. An ASD with partial anomalous pulmonary venous drainage is another common combination of congenital heart defects. The Warden procedure to reestablish pulmonary venous connection to LA with one self-made interior pipeline is feasible with the TVTCS technique [53].

### 3.3 Cardiac Mass

Left atrial myxoma is the most common cardiac mass which can be performed with TVTCS [54]. Assessing the anatomy characteristics of the cardiac vasculature is crucial before considering the procedure to remove a cardiac tumor. The most common tumor is the pedicled cardiac atrial myxoma which can be performed with TVTCS [55]. For other unknown cardiac masses, cardiac contrast echocardiography is suggested to be performed to distinguish the tumor mass from a venous thromboembolism. Contrast-enhanced CT could further provide the information on the size, location of the cardiac mass, and the infiltration of the mass onto the adjacent tissue, such as is seen with a primary lymph tumor [56]. Brain MRI is a valuable tool to recognize metastasis from a primary lung tumor to the left atrium. The port-access approach is not indicated unless the biopsy is only the purpose for the procedure [57].

### 3.4 Hypertrophic Obstructive Cardiomyopathy (HOCM)

Septal myectomy resulted in superior long-term survival outcomes than interventional alcohol septal ablation for the patients with HOCM [58]. The trans-mitral approach via TVTCS is relatively safe for elderly patients above 60 years [59]. The TVTCS approach not only facilitates the exposure of the ventricular septum, mitral valve, and sub-valvular apparatus from the visual angle of cardiac cavity, but also enables the surgeons to perform septal myectomy in complex cases like mid-ventricular obstruction and concomitant mitral valve interventions for anterior mitral leaflet extension [60]. The relief of pressure gradient on left ventricular outflow tract quantified with left ventricular outflow tract velocity and interventricular septal thickness by TEE was the therapeutic emphasis for left ventricular septal myectomy [61]. Postprocedural mitral regurgitation usually suggests an extended myectomy rather than prothesis implantation because of the heterogeneous morphology on the left ventricular outflow tract and the not uncommon coexistence of a congenital mitral valve apparatus anomaly [62].

Cardiac CT is highly valuable and versatile tool which could identify the components of outflow tract obstruction and apical aneurysm [63]. MRI showed LA reverse remodeling in HOCM patients after septal myectomy, which displayed a reduction in LA size and a restoration in LA reservoir and function, but unchanged LA conduit function [64]. However, myocardial fibrosis was increased after myectomy in the one-year follow-up, which is observed by late gadolinium enhancement in the left ventricle [65].

### 3.5 Atrial Fibrillation

Patients with nonvalvular atrial fibrillation (AF) are strongly recommended in expert guidelines to receive an ablation procedure for restoration of sinus rhythm including a Cox-Maze surgical procedure when there is a contraindication for oral anticoagulation [66]. For a single ablation procedure without intracardiac disease, TVTCS is a more comfortable and economic approach than MS. Complete occlusion of the LAA could be safely achieved through epicardial LAA clipping by TVTCS [67]. The closure device should be specifically designed for the appendage to guarantee efficacy and safety and to optimize surgical placement on the beating heart [12]. The residual LAA stump of less than 10 mm after the procedure measured by cardiac CT is considered to be clinically safe [68]. The reduction in flow velocity within the LAA is associated with imaging features on volume and filling defects in patients with AF, which indicates the risk of thromboembolic events [69]. A total of three ablation lines are suggested for pulmonary vein antral clamping during TVTCS for avoiding unnecessary repetitions [70]. The combined method using surgical and interventional approaches aims to provide transmural conduction blockade by replicating Cox-Maze IV lesions for the refractory cases [71].

### 3.6 Aortic Valve Diseases

Aortic valve replacement with TVTCS has an advantage that the stended bioprostheses does not require suture deployment. Isolated aortic valve replacement via TVTCS has been performed since 2013, first reported by Cresce *et al*. [72] but has been associated with a high complication rate such as a new permanent pacemaker implantation. Endoscopic aortic valve replacement concomitant with combined procedures was safely administered by the same surgical group with acceptable complication rates [73]. Yilmaz *et al*. [74] reported a higher re-exploration rate (2.6%) and mid-term mortality at three-year follow-up (7.5%). Two-port approach was also successfully performed on aortic valve diseases by TVTCS in recent years [75]. The approach should be cautioned in the presence of calcific aortic valve stenosis because the excision is located adjacent to the coronary ostium, and embolic debris and coronary dissection can lead to the difficulty in weaning from CPB and concomitant salvage coronary artery bypass grafting may be necessary [76].

## 4. Comments

TVTCS had an advantage over MS in terms of smaller incisions, convenient preparation, and high-resolution. These benefits were highly evident in the context of emergency, or inaccessible cases like re-do mitral valve surgery compared to the conventional approach. Successful conduction of TVTCS is based on the external conditions including a broad thoracic cavity and effective peripheral cannulation after adequate preparation and systemic evaluation, and performed by a qualified cardiac surgeon trained in TVTCS.

MCI not only achieves the needs for cardiac imaging examinations but also helps to prepare the working conditions for TVTCS. Over the past two decades, MCI is gaining popularity for CPB planning and port placement for TVTCS. It results in more precise information than can be obtained with traditional manual estimates. MCI provides quantitative or qualitative valuation for the specific parameters needed to ensure the successful administration of TVTCS and result in a smooth recovery. These measurement tools include chest X-ray, CT, CTA, MRI, Cardiac catheterization, Doppler ultrasonography, TTE, TEE, and contrast UCG. Not one single imaging mode can provide the whole scope of images and information needed for TVTCS. The integrative information from MCI results in a risk assessment algorithm which is useful to mitigate the risk of adverse events. By virtue of this data, the scope of conducting TVTCS for cardiac surgical diseases are broadened from ASD and AF in earlier years, to atrioventricular valve repair or replacement to trans-mitral septal myectomy in recent years. Some urgent procedures can now also be managed with TVTCS to mitigate the risk of adverse effects with the use of these minimally invasive techniques.

TVTCS is one such procedure in which complications such as cannulation failure, secondary injury, and device dysfunction can be decreased substantially with incorporation of physician driven imaging and digital tools. Patients benefit from preprocedural cardiac CT reconstruction and 3D printing, and intraprocedural 3D echocardiography and dynamic fusion imaging. The clinical practice of TVTCS has benefited not only from individual imaging technical progress, but also from fusion technology from multiple imaging modes, including computational modeling and mobile detection modes [77]. Overcoming the barriers stemming from data heterogeneity among the sorts of detection instruments to establish the common standard for clinicians is a clinical challenge [78]. A realistic stereoscopically anatomical model from the visible imaging datasets permits the developments of custom-tailored procedure strategies.

Coupling of the imaging requirements is conducive to excluding the potential frustrations when choosing TVTCS, which contributes to an effective and accurate comprehensive prediction system for the manipulation of TVTCS. Thus, standardized imaging monitoring in addition to individualized protocol adoption should be advocated according to the characteristic of the subjects. Integration of MCI technologies into the process of diagnosis and operation is widening the accuracy and scope of conducting the TVTCS techniques for cardiac surgical diseases.

## 5. Conclusions

In conclusion, an understanding of MCI, individually and collectively, affect the diagnosis and intervention of the specific details of cardiac structural diseases could lead to a better appraisal of the applicability and safety of TVTCS and aid in the conception of new preventive and therapeutic strategies to limit the predictable risk of bleeding and failure especially under unfamiliar circumstances. The findings from MCI should be incorporated into clinical protocols to provide decision-making strategy for the patients considering TVTCS strategies. Future directions on MCI in the field of TVTCS should be developed into formation of the optimized custom-tailored procedure strategy on the basis of computational modeling prediction and artificial intelligence assistance which is implanted in realistic anatomical characteristics from the series of visible imaging datasets.
